# The case for bilateral mastectomy and male chest contouring for the female-to-male transsexual

**DOI:** 10.1308/003588413X13511609957290

**Published:** 2013-03

**Authors:** C Richards, J Barrett

**Affiliations:** West London Mental Health NHS Trust, UK

**Keywords:** Mastectomy, Transsexualism, Transgender, Funding, Trans man

## Abstract

**Introduction:**

In the UK, funding for a bilateral mastectomy (BLM) and associated chest recontouring for female-to-male transsexuals (trans men) has been rejected by some funding authorities on a number of unsustainable grounds.

**Methods:**

As funding is increasingly an important area for both surgeons and referrers, we undertook a review of the small amount of literature pertaining to this issue and considered it in light of our clinical experience of this group.

**Findings:**

The literature showed that BLM is necessary for trans men to live safely and effectively in their reassigned gender role, and further that it acts as a prophylaxis against distress, ameliorates extant distress as well as providing improved quality of life and global functioning for this patient group.

Trans men are those people who are assigned female at birth but who live in a male role. To this end, trans men often seek surgical and hormonal assistance to present in the role that best reflects their internal sense of being male. This may include hormonal manipulation to induce beard and body hair growth, greater musculature, the cessation of menses and, possibly, capital hair loss. There will certainly be a change of social role, including name and documentation, as well as re-establishing occupational and social relationships in the male role.

Trans men may also wish to have genital surgery to approximate male genitalia. However, along with the wish to live socially in a male role, the overriding need to be rid of developed secondary sexual characteristics (in the case of trans men, breasts) is the *sine qua non* of the diagnosis of gender identity disorder,^1^ also known as transsexualism,^2^ most probably owing to the fact that these are such a marked signifier of femininity.^3–5^

In order to rid themselves of the appearance of female breasts, trans men often ‘bind’ them with bandages or special breast binders in order to approximate a male chest contour. However, this is impractical if they have large breasts, can be uncomfortable in hot weather, can cause back pain and causes poorer results in any BLM procedures that may be undertaken due to a ptosis of the breast.^6–8^

In addition, it is, of course, not possible for trans men who have not had a BLM to go bare chested in order to swim, to get changed in front of other men or be sexually intimate. Hage states: ‘The wish to re-enter society as a person with the physical and mental gender of choice without being “spotted” or without anyone “knowing” fulfils almost all of the preoperative objectives of the transsexual and should also be the goal of surgical treatment.’^9^ Monstrey *et al* state: ‘The first and arguably most important surgical procedure performed in the female-to-male transsexual is the creation of an aesthetically pleasing male chest.’^8^

Consequently, it is standard clinical practice in the UK to consider a BLM if the patient has requested this and it is deemed psychologically appropriate by a specialist,^10,11^ who should be from a ‘reputable gender team’^9^ or a ‘well coordinated multi-disciplinary team of professionals’.^12^ Hage and Bloem state that BLM will enable the female-to-male transsexual ‘to live as a man both in public and in private, thus facilitating the adjustment to a male lifestyle’.^6^

Furthermore, according to Newfield *et al*, chest reconstruction ‘not only enhances the [female-to-male] tans-gender identity, increases self esteem, and improves body image, but provides some security and safety for those who remove their shirts in public areas’.^13^ Monstrey *et al* concur, suggesting a trans man’s BLM ‘greatly facilitates the real-life test [the period of time a trans man must live in his preferred gender role prior to being considered for genital surgery, now commonly called the real-life experience in recognition of the fact that the man is not being tested but rather is coming to terms with the reality of his decisions] or his adjustment to a male life style’ and that obtaining ‘a male chest contour is of utmost importance for [female-to-male] transsexuals’.^12^ This is the case in the great majority of trans males and is in line with standard practice in a variety of other countries^14–16^ as well as the international guidelines of the World Professional Association for Trans-gender Health^17^ (formerly the Harry Benjamin International Gender Dysphoria Association).^18^

Unfortunately, while some funding authorities recognise the necessity of BLM for the reasonable functioning of trans men both at work and beyond (and so regard it as a ‘core procedure’ with protected funding), others consider it to be a purely cosmetic procedure, citing as a justification the fact that they are not aware of the evidence that shows that BLM assists the lives of trans men to a marked extent. We therefore undertook a search of the small amount of extant literature pertaining to this complex topic through snowballing the literature available. This was then integrated with clinical experience to give the overview of the necessity for BLM, which forms this paper and addresses this lacuna in the literature.

## Exceptionality

Drawing on clinical experience, if we consider a hypothetical trans man with a beard, a neophallus (ie a surgically contructed penis), a role in a construction company, and a full pair of female breasts, it should be apparent that BLM is necessary for the avoidance of psychopathology. Psychopathology in these cases is associated with factors that make it difficult for trans people to be accepted in their gender of presentation and remind them of their transsexualism.^19^ In trans males, the narrower natal female chest makes the breasts more apparent than for the natal male, making the case for mastectomy and chest recontouring arguably more marked for trans men than that in the case of gynaecomastia in the natal male. This means that trans men can be considered exceptional in this regard.

Furthermore, trans men have a variety of cues to their natal gender that may not be present in natal males. For example, they are often of shorter stature, are finer boned, have smaller hands and feet, have some residual female socialised mannerisms, all of which mean that the addition of a marked female cue such as feminine breasts risks making their trans status more apparent, so putting them at raised risk of discrimination or harm on a day-to-day basis.^20,21^ The difficulty of hiding the chest, in contrast to the genitalia, in order to effect a coherent masculine presentation and to avoid possible harm may be one reason why some trans men *only* wish to have chest surgery, and choose to consider possible genital surgery at a later date or not at all.^18^ We consider it paradoxical, therefore, that some funding authorities only fund genital and not chest surgery.

## Choice

There has been some assertion previously that transsexualism is a choice made by the transsexual patient and that, as such, it should be secondary in funding arrangements to other priorities. Of course, the National Health Service (NHS) does fund surgery for conditions clearly brought about by matters of choice (eg smoking and some forms of obesity). However, in the case of transsexual individuals, evidence is mounting that there is a biological aetiology affecting neurological development over which trans people have no control.^22–28^

It is for this reason that mandatory psychological interventions have failed repeatedly^29–31^ and, indeed, have proven to be harmful.^32,33^ Thus, as the mind may not be altered in line with the body, the body must be altered to be in line with the mind.^10,29,34–35^ As a result, we concur with Niewfield *et al* that: ‘[Female-to-male] transgender people are requesting services that are not incidental, cosmetic remedies, but rather therapies key to their well being.’^13^

## Distress and dysfunction

In addressing the funding implications of BLM for trans men, two matters are often considered: first, does the surgery alleviate distress or act as a prophylaxis against distress? Second, does the surgery improve quality of life, social, psychological or occupational functioning? NHS care is usually based only on the former and we would question any higher requirements for trans people than other patients in this regard. Nevertheless, the literature shows that chest surgery for trans men meets both requirements.

It is worth noting that transsexualism is a fundamentally subjective experience. There are no diagnostic tests that are independent of the patient’s reported sense of self.^10,35^ This means that all interventions are aimed at ameliorating patient distress concerning their sense of their self in the world in the wrong gender (or prophylaxis against such distress). Any outcome measures will therefore necessarily be reflective of the subjective sense of amelioration (or otherwise) of distress. Consequently, Kuhn *et al* state that ‘an evaluation of sexual reassignment surgery (SRS) [including BLM] can only be made on the basis of subjective data because SRS is intended to solve a problem that cannot be determined objectively’.^36^

We argue that it is disingenuous to refuse funding on grounds of lack of empirical support if the only empirical support that will be accepted is that which *necessarily* cannot be found due to all possible evidence being subjective. Much evidence has been found, however, that shows that patient distress is ameliorated and, further, that better outcomes are found after BLM. For example, better patient functioning has been reported in the literature^6–9,12,37,38^ and better occupational functioning has been reported explicitly.^39^ Moreover, improved quality of life has been reported.^13,36^

Newfield *et al* interviewed 376 trans male participants and scored them on the Short Form 36 health survey (version 2).^13^ They found that those who had received BLM reported higher quality of life scores than those who had not received surgery, with statistically significant results (*p*<0.01) for the general health and social functioning scales as well as three of the mental health measures. They also found that, when controlling for income and education, this held for the general health domain, suggesting that, for trans men, the ability to earn a living is most important to their quality of life. Unfortunately, in the UK, this may be affected adversely by being exposed as trans at work,^21^ increasing the need for trans men to receive interventions such as chest surgery that may protect against this.

## Conclusions

We recommend the funding of trans men for BLM not only on the basis of our extensive clinical experience in the largest gender identity clinic in the world^40^ but also on the basis that the literature supports our assertion that surgery for these patients alleviates distress and improves functioning. Well selected patients regret BLM surgery extremely rarely;^6,12,36,39^ and, although some studies suggest that quality of life for trans men is reduced *both before and after* surgery compared with the general population, there is nevertheless an improvement *from* before *to* after when BLM is undertaken. A fairer comparison is with people who have had genital surgery for a congenital problem – and trans male quality of life is in fact in line with people who have had surgery for hypospadias, who also do better afterwards.^41^

While we recognise that funding bodies are operating within strict budgetary limits, we caution that delays in funding will cause distress, which carries with it the associated costs of amelioration through supportive psychotherapy during the wait, ongoing assessment and, in extreme cases, hospitalisation. In addition, breast skin that has become inelastic due to prolonged binding can alter the surgeon’s choice of technique,^6–8^ which itself may have associated cost implications. Overall costs to funding authorities may therefore be reduced by timely surgical treatment for this patient group.
